# Assessment of Qatar community pharmacists’ competence and practices related to renal and gastrointestinal adverse effects of nonprescription NSAIDs

**DOI:** 10.1016/j.jsps.2022.06.011

**Published:** 2022-06-22

**Authors:** Yaw Boahene Owusu, Wishah Hamza Elkhalifa, Ahmed Awaisu, Nadir Kheir

**Affiliations:** a Department of Clinical Pharmacy and Practice, College of Pharmacy, QU Health, Qatar University, Doha, Qatar; b Pharmacy Department, Hamad General Hospital, Hamad Medical Corporation, Doha, Qatar; c Department of Clinical Sciences, College of Pharmacy and Health Science, Ajman University, Ajman, United Arab Emirates

**Keywords:** Community pharmacist, NSAIDs, Renal adverse effects, Gastrointestinal adverse effects, Knowledge, Attitude

## Abstract

**Introduction:**

Non-steroidal anti-inflammatory drugs (NSAIDs) are among the most frequently dispensed nonprescription drugs in community pharmacies. However, inappropriate use of NSAIDs by consumers has been associated with development of gastrointestinal (GI) injuries and renal injuries. Community pharmacists’ education of consumers on proper use of NSAIDs and their associated adverse effects has been shown to reduce the GI and renal injuries. In Qatar, no studies have been done to assess the community pharmacists’ knowledge, attitude, and practices related to renal and GI adverse effects of NSAIDs. Therefore, this study aimed to assess Qatar community pharmacists’ knowledge, attitude, and practices on the safe use of nonprescription NSAIDs to reduce the risk of kidney and GI injuries.

**Methods:**

A cross-sectional web-based survey was conducted among community pharmacists in Qatar. A pre-tested 28-item questionnaire that was developed through a multi-phase iterative process was administered to a convenient sample of community pharmacists in Qatar. Data were analyzed using descriptive and inferential statistics with statistical significance set at p < 0.05.

**Results:**

Overall, 114 community pharmacists responded to the online questionnaire (response rate 15.2%). Approximately 90% of the community pharmacists demonstrated from good to excellent knowledge on the renal and GI adverse effects of NSAIDs, with none of their sociodemographic and professional characteristics having a significant effect on their knowledge scores. More than half of the pharmacists reported that they always or usually educated patients on the dosage (98.6%), administration (95.8%), side effects and precautions (78%), and contraindications (71.2%) of NSAIDs during their routine practices. The majority of the pharmacists had positive attitude towards educating patients about adverse effects of NSAIDs, as well as identifying high-risk patients who should avoid nonprescription NSAIDs. However, 45.7% of the pharmacists strongly agreed or agreed that educating patients about NSAIDs can be time consuming.

**Conclusion:**

Community pharmacists in Qatar demonstrated good knowledge of the renal and GI adverse effects of NSAIDs with some obvious areas of improvement, and this can be reinforced through continuing professional development. They also showed positive attitudes towards protecting patients against the renal and GI adverse effects of NSAID. However, a significant proportion of the pharmacists admitted that educating patients on NSAIDs was time consuming, which is a cause of concern warranting further investigation. Community pharmacy managers should provide community pharmacists adequate time and support to educate individuals at risk of renal and GI injuries who obtain NSAIDs from their pharmacies. Also, the Ministry of Public Health of Qatar should consider making counseling on high-risk medications (e.g., NSAIDs and insulin) by community pharmacists mandatory so that measures can be put in place in the pharmacies to free the pharmacist for education and counseling.

## 1 Introduction

Over-the-counter (OTC) non-steroidal anti-inflammatory drugs (NSAIDs) are used extensively worldwide to self-manage pain, inflammation, or fever associated with various symptoms and disease conditions. For example, in the United States of America (USA), 39.7% of households purchased ibuprofen as an OTC NSAID in 2017 (Nielsen, 2018). However, NSAIDs in general can cause serious harms to body organs or systems if used inappropriately. Gastrointestinal (GI) injury (e.g., dyspepsia, upper GI bleeding, peptic ulcer disease, and life-threatening GI perforation), and acute renal injury (e.g., interstitial nephritis) are among the most common serious adverse events resulting from inappropriate NSAIDs use (Oliviu, 2017). These potentially fatal adverse events of NSAIDs are attributed to taking higher than the recommended dose, utilization for a longer duration than recommended, and therapeutic duplication (i.e. taking multiple NSAIDs at the same time). A one-week online diary study conducted on 1,326 ibuprofen users in the USA reported that 37% of the ibuprofen users also used another NSAID during the same week (Kaufman et al., 2018). Another study suggests that users of OTC NSAIDs were generally unaware of their potential adverse effects, implying the possibility of inadequate safety education from prescribers and pharmacists (Wilcox et al., 2005). Furthermore, physicians who educate patients on adverse drug reactions (ADRs) of NSAIDs are more likely to focus on the GI harms than the kidney-related harms, as shown in a study of orthopedic doctors who frequently prescribed NSAIDs (Phueanpinit et al., 2017).

In the presence of underlying risk factors for GI and renal injuries, the risk of NSAIDs-related injuries is elevated further. These risk factors include advanced age (>60 years), previous history of ulcer, concomitant use of an oral anticoagulant or corticosteroid, use of more than one NSAID, and presence of comorbid conditions such as renal and hepatic failure (Vonkeman and van de Laar, 2010, Harirforoosh et al., 2013). In many developing countries, the public can buy a wider range of nonprescription NSAID products, predisposing even the well-informed high-risk individuals to fatal injury or organ damage.

The community pharmacy setting is a safety net for patients or customers who self-medicate with OTC NSAIDs. The community pharmacist is often the first port of call and the most accessible healthcare professional who provides free consultation services and professional advice (ISMP, 2007). Therefore, they have a professional responsibility to educate customers and patients about safety-related issues, adverse effects, and precautions to minimize risks of harm when they dispense nonprescription NSAIDs. For individuals with absolute contraindications to the use of NSAIDs or are at high risk of GI and kidney injuries, the community pharmacist needs to provide them with alternative evidence-based treatment options. For this reason, pharmacists should be able to appropriately screen individuals for risk factors that increases their chances of harm from NSAIDs use. A study conducted in Thailand showed that 95.5% of community pharmacists self-reported regularly screening for risk factors when dispensing non-selective NSAIDs, while 63.2% regularly communicated adverse drug reaction (ADR) information (Phueanpinit et al, 2018). A similar study in Saudi Arabia found that 84.7% of community pharmacists regularly or occasionally screened for risk factors when dispensing non-selective NSAIDs, while having more than five years of practice experience or additional pharmacist on the same shift significantly increased the likelihood of counseling about ADRs (Malebari et al, 2020). In order to educate the public about NSAIDs, the community pharmacist should have adequate knowledge about the pharmacology and therapeutics of NSAIDs, as well as possess positive attitude towards educating their customers on their safe use.

In Qatar, anyone can obtain a wide range of NSAIDs from community pharmacies without a prescription. Yet, the prevalence of NSAIDs use is poorly documented in Qatar even though it is widely dispensed in community pharmacies. A significant proportion of the population of Qatar are laborers engaged in manual labor who regularly buy NSAIDs from community pharmacies as a result of occupation-related musculoskeletal pain. Similarly, sedentary workers are also likely to utilize NSAIDs regularly for chronic and lower back pain. A large cross-sectional study conducted in the primary care setting on a representative sample of non-manual workers and unemployed patients reported high prevalence of low back pain of 59.2% in Qatar (Bener et al, 2013).

It is a professional obligation for the community pharmacist to ensure NSAIDs dispensed to customers will not cause harm or fatal organ injury. Educating patients in the community pharmacy on NSAIDs avoidance have been shown to be an effective practice in reducing acute kidney injuries in high-risk groups (Jang et al., 2014). Similarly, community pharmacists’ interventions reduce the risk of NSAID-related gastropathy and other GI-related side effects in high-risk patients (Teichert et al., 2014, Ibanez-Cuevas et all., 2008). Overall, there is limited literature internationally and regionally on the competencies, attitude, and practices of community pharmacists related to the safety of non-prescription NSAIDs. Furthermore, in Qatar, there are no guidelines for community pharmacists to follow to ensure NSAIDs are dispensed safely to consumers. Therefore, this study aimed to assess community pharmacists’ knowledge, practices, and attitude on the safe use of nonprescription NSAIDs to reduce the risk of renal and GI injuries in Qatar.

## 2 Methods

### 2.1 Study design and participants

This was a cross-sectional questionnaire-based study that was conducted between October 2016 and May 2017 in community pharmacies in Qatar. Licensed community pharmacists were e-mailed the link to the survey via SurveyMonkey®. In addition, the pharmacy managers of the three largest pharmacy chains in Qatar (i.e., Care n Cure, Kulud, and Wellcare Pharmacies) and one independent pharmacy (i.e., Ebn Sina) were contacted to disseminate the survey link to their pharmacists.

### 2.2 Sample and sampling technique

A minimum sample size of 255 was calculated with the Raosoft® online calculator using the following parameters: 5% margin of error, 95% confidence interval, estimated number of community pharmacists of 750, and estimated response distribution of 20% (Raosoft® Sample Size Calculator, 2016). Qatar is a small nation of 2.8 million people; therefore, there are a relatively fewer number of registered community pharmacies and pharmacists to use as sampling frame. Email addresses of community pharmacists were obtained from the Qatar Council of Healthcare Practitioners (QCHP), a department of the Ministry to Public Health of Qatar that licenses all practicing pharmacists in Qatar. The research team sent out the survey to 750 valid email addresses of licensed community pharmacists. In order to maximize the response rate, and get a representative sample of community pharmacists, the research team contacted the pharmacy managers of the three largest pharmacy chains in Qatar (i.e., Care n Cure, Kulud, and Wellcare Pharmacies) and one independent pharmacy (i.e., Ebn Sina) to send the survey link to their pharmacists.

### 2.3 Survey instrument development and validation

The 28-item questionnaire was created and validated in English only. The three composites of the questionnaire assessed the current knowledge of the community pharmacists on NSAIDs-related kidney and GI injuries; current practices on proper use of NSAIDs and; attitudes towards NSAIDs use in Qatar. The items of the questionnaire were adapted and modified from two previous studies (Pai, 2015, Grimmer et al., 2002). The developed questionnaire was evaluated for content validity (adequacy of items, comprehensiveness, type, flow, and clarity of items) by four pharmacy educators with experience in pharmacy practice-based research and survey instruments development. Appropriate changes, including, reducing the number of knowledge items from 14 to 10, and changing short answer questions to multiple-choice questions were made to the questionnaire based on the feedback received from the experts through an iterative process among the research team members. Furthermore, the version of the questionnaire that was agreed to be appropriate for use in the study underwent face validity (test for clarity, understanding/readability, ambiguousness of items, and questionnaire completion time) through pre-testing among three practicing community pharmacists and four recent pharmacy graduates. Minor changes such as merging two items each in the practices and beliefs domains were made based on the feedback received through the piloting process. Overall, few revisions were made to the originally adapted survey by the research investigators through an extensive iterative process to address the issues identified during the validation process and to ensure its appropriateness for the study.

The final version of the questionnaire contained 28 items, divided into three composites (22 items) and a section on demographics (6 items). The knowledge composite contained nine multiple-choice questions and one true or false question. Therefore, the maximum obtainable score for this composite was 10 points. The current practice composite comprised nine questions related to patient education on safe use of NSAIDs, as well as the community pharmacist’s professional development endeavor related to NSAIDs. Lastly, the composite of attitudes towards NSAIDs use comprised three items measured on a 5-point Likert scale focusing on the attitudes of community pharmacists regarding counseling patients and customers on NSAIDs-related kidney and GI side effects as well as the importance of the community pharmacist’s role in today’s practice.

### 2.4 Survey administration

SurveyMonkey®, an online survey tool was used to administer the anonymous survey. The survey link was emailed to 750 valid email addresses of licensed community pharmacists in QCHP database, as well as through the managers of the largest chain community pharmacies in Qatar. Reminders were sent out to the community pharmacists by email and through pharmacy managers at least every two weeks to encourage more participation during the six-month period the survey link remained active.

### 2.5 Data analyses

Data collected via the SurveyMonkey® responses were exported into IBM’s Statistical Package for Social Sciences (SPSS) version 23 software (IBM, Armonk, NY, USA). Both descriptive and inferential statistical analyses were applied. Total knowledge score was calculated for each respondent and categorized as poor (0–4 points), good (5–6 points), very good (7–8 points), and excellent (9–10 points). Since the possible knowledge score ranges from 0 to 10 points, the scores below the 50th percentile (0–4) were categorized as poor, and scores equal to or greater than the 50th percentile were categorized as good, very good or excellent. Mann-Whitney U and Kruskal-Wallis tests were used to analyze respondents’ knowledge score based on their demographic and professional characteristics. In addition, the Chi-square test was used to determine the influence of demographic and professional characteristics on the knowledge score categories (i.e., poor, good, very good and excellent). A p-value of < 0.05 was used to determine statistical significance.

### 2.6 Ethics approval

The study protocol, questionnaire, and informed consent form were reviewed and approved by the Qatar University Institutional Review Board (approval number: QU-IRB 687-E/16).

## 3 Results

### 3.1 Socio-demographic and professional characteristics of the respondents

Over a six-month period, a total of 114 community pharmacists responded to the online questionnaire, achieving a response rate of 15.2% (114/750), of the population of community pharmacists practicing in Qatar who received the online survey link through email. Of the 114 that responded, 34 completed only the socio-demographic and professional characteristics without completing the remaining parts of the questionnaire. Therefore the usuable rate was 70.2% (80/114).

Details regarding the community pharmacists’ socio-demographic and professional characteristics are provided in Table 1. The majority of the community pharmacists who responded to the online survey (63/80, 78.8%) were males (Table 1). More than half of the respondents were aged 30–39 years **(**46/80, 57.5%), from North Africa (39/80, 48.8%), and East Asia (34/80, 42.5%). More than half of the respondents (44/80, 55.7%) got their license to work as community pharmacists in Qatar less than five years prior to completing the questionnaire. The majority of the community pharmacists (54/80, 68.4%) last updated their knowledge on NSAIDs within one year prior to completing the questionnaire. The highest degree obtained by most of the community pharmacists practicing in Qatar (67/80, 83.8%) was bachelor’s (BSc) degree.

**Table 1:** Demographic and professional characteristics of community pharmacists practicing in Qatar (*n* = 80).

Characteristic	Frequency (%)
**Gender**	
Male	63 (78.8)
Female	17 (21.2)
**Age**	
<30 years	26 (32.5)
30–39 years	46 (57.5)
≥40 years	8 (10)
**Nationality**	
GCC country (Qatar, Oman, Bahrain, Emirates, Saudi Arabia, Kuwait)	1 (1.3)
Middle Eastern country other than GCC (Jordan, Palestine, Lebanon, Syria, Iraq, Yemen)	6 (7.5)
North African country (Tunisia, Egypt, Sudan, Morocco, Algeria, Libya)	39 (48.8)
Asian country (Iran, Pakistan, India, Bangladesh, The Philippines, etc.)	34 (42.5)
Duration of practicing as licensed community pharmacist in Qatar*	
<5 years	44 (55.7)
5–10 years	24 (30.4)
greater than10 years	11 (13.9)
When did you last update your knowledge on NSAIDs?*	
I did not update my knowledge	3 (3.8)
<1 year ago	54 (68.4)
1–5 years ago	18 (22.8)
6–10 years ago	1 (1.3)
I do not recall	3 (3.8)
**Highest pharmacy degree**	
Bachelor’s degree	67 (83.8)
Master’s degree	9 (11.3)
Doctor of Pharmacy	3 (3.8)
Other#	1 (1.3)

*Missing data.

# Other: Postgraduate Diploma in Pharmacy.

### 3.2 Current knowledge of community pharmacists on the renal and gastrointestinal adverse effects of NSAIDs

Table 2 provides the descriptive statistics of the knowledge scores. The distribution of the total knowledge scores of the surveyed community pharmacists ranged from 1 to 10. The mean ± SD total knowledge scores among the community pharmacists was 7.2 ± 2.1 and the median (IQR) was 7 (3), corresponding to good knowledge (Table 2). A total of 80 community pharmacists solved the knowledge test, out of which 30% (24/80) scored 9 to 10 points (excellent), 37.5% (30/80) scored 7 to 8 points (very good), 22.5% (18/80) scored 5 to 6 points (good), and 10% (8/80) scored from 0 to 4 points (poor). Table 3 illustrates the community pharmacists’ performance on individual knowledge items related to NSAIDs-associated renal and GI adverse effects. The knowledge question that the highest proportion of the community pharmacists answered correctly (66/79, 83.5%) was a true/false question that asked “Unlike systemic NSAIDs, topical NSAIDs have lower risk of causing epithelial injury in the gastrointestinal tract.” Similarly, the question that a large proportion of the community pharmacists answered incorrectly (37/80, 46.3% answered incorrectly) was “NSAID interact with all of the following drugs except: 1-Diuretics 2- Clopidogrel 3- Warfarin 4- Albuterol.”.

**Table 2:** Summary statistics of community pharmacists’ knowledge scores distribution (*n* = 80).

Knowledge score	Values
Minimum	1
Maximum	10
Mean ± SD	7.2 ± 2.1
Median (IQR)	7 (3)
Knowledge score category (Range)	Frequency (%)
Poor (0 – 4)	8 (10)
Good (5 – 6)	18 (22.5)
Very good (7 – 8)	30 (37.5)
Excellent (9 – 10)	24 (30)

**Table 3:** Current knowledge of community pharmacists on NSAIDs associated renal and gastrointestinal adverse effects (*n* = 80).

Knowledge item	Correct option	Number of responses	Correct response n (%)
Q1: NSAIDs are indicated in all of the following conditions except:	Chronic gout	80	56 (70)
Q2: NSAIDs interact with all of the following drugs except:	Albuterol	80	43 (53.8)
Q3: NSAIDs are contraindicated in all of the following conditions except:	Osteoporosis	80	64 (80)
Q4: Which of the following is a gastrointestinal side effect of NSAIDs:	Upper gastrointestinal bleeding	80	63 (78.8)
Q5: Unlike systemic NSAIDs, topical NSAIDs have lower risk of causing epithelial injury in the gastrointestinal tract:	True	79	66 (83.5)
Q6: Inhibition of prostaglandin synthesis causes:	Increased gastric acid secretion, decreased bicarbonate secretion and decreased mucus secretion	79	53 (67.1)
Q7: Risk factors for developing gastric injury include all of the following except:	Age < 65 years	79	55 (69.6)
Q8: The risk of NSAID-induced kidney injury increases with:	Concomitant use of ACE inhibitors	75	61 (81.3)
Q9: All the following patients should avoid self-medication with NSAIDs except:	Patients with hypothyroidism for which they take levothyroxine orally	78	62 (79.5)
Q10: Which of the following is the most appropriate alternative for a patient at high risk for NSAID induced acute kidney injury after an acute musculoskeletal injury?	Acetaminophen/Paracetamol	80	51 (63.7)

### 3.3 Influence of community pharmacists' characteristics on their knowledge

Further analysis of the results were done to assess the influence of the pharmacists’ characteristics on their knowledge score. The inferential analyses (Mann-Whitney *U* test and Kruskal-Wallis test) showed that none of the characteristics (gender, age, nationality, place of practice, duration of obtaining license, highest pharmacy degree) had a significant effect on the knowledge score (i.e., p˃0.05). In terms of age, those in the age ranges of 30–39 years and 40–50 years age groups seemed to be more knowledgeable with median (IQR) scores of 8 (2) and 9 (2) respectively. Respondents from a Middle Eastern country had a higher median (IQR) score of 9 (3) in comparison to those from a North African country, 8 (3) or an Asian country, 7.5 (3). The median (IQR) score for pharmacists who obtained their licenses more than 10 years prior to the questionnaire administration, 9 (3) was higher than those who obtained their licenses between 5 and 10 years, 8 (3) and less than 5 years before completing the questionnaire, 7.5 (3). In addition, the median (IQR) score for pharmacists whose highest professional degree in pharmacy is BSc, 8 (2) was higher than those with master’s degree, 7 (4).

### 3.4 Community pharmacists’ practices on the safe use of NSAIDs to reduce the risk of renal and gastrointestinal injuries

**Fig. 1:**
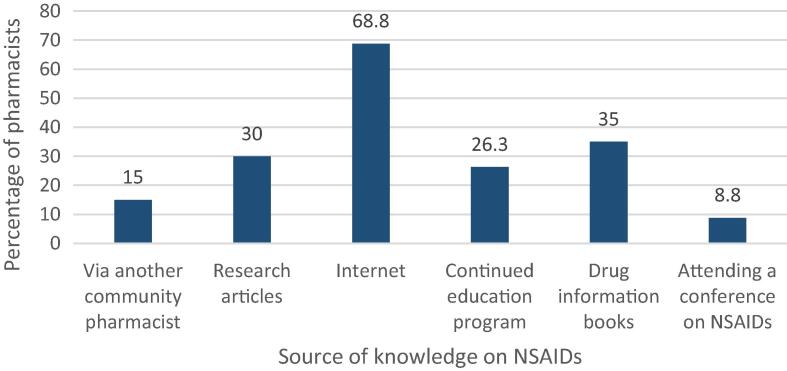
Sources used by community pharmacists to update their knowledge on NSAIDs (*n* = 80).

Most of the community pharmacists surveyed (54/79, 68.4%) stated that they had updated their knowledge on NSAIDs within a year prior to completing the questionnaire, while 22.8% (18/79) updated their knowledge on NSAIDs one to five years prior. Of those who updated their knowledge, 68.8% (55/80) used online resources, 35% (28/80) used drug information books, 30% (24/80) used research articles, 15% (12/80) through another community pharmacist and 26.3 % (21/80) attended a continuing education program to update their knowledge on NSAIDs (Fig. 1). Only seven of the community pharmacists (8.8%) updated their knowledge on NSAIDs by attending a conference. Of those surveyed, around 69% (49/71) tend to recommend diclofenac to their customers, while 26.8% (19/71) recommend ibuprofen most commonly.

The frequency with which community pharmacists provided education to patients/customers on the instructions of taking the medicine, dosage, side effects/precautions, and contraindications are summarized in Table 4. According to the results presented, the majority of the community pharmacists (65/73, 89%) always educated patients on how to take the medicine, and 90.4% (66/73) always educated patients on the dosage. On the contrary, less than half of the community pharmacists (35/73, 47.9%) always counseled patients on side effects/precautions and approximately 37% (27/73) always counseled patients on NSAID’s contraindications (Table 4).

**Table 4:** Practice in relation to community pharmacists in Qatar educating patients on how to take NSAIDs safely and adverse effects of NSAIDs (*n* = 80).

During patient counseling, how frequent do you educate patients on: Instructions on taking the medicine*	**Frequency (%)**
Always	65 (89)
Usually	5 (6.8)
Sometimes	1 (1.4)
Never	2 (2.7)
During patient counseling, how frequent do you educate patients on: Dosage*	**Frequency (%)**
Always	66 (90.4)
Usually	6 (8.2)
Sometimes	1 (1.4)
Never	0 (0)
During patient counseling, how frequent do you educate patients on: Side effects and precautions*	**Frequency (%)**
Always	35 (47.9)
Usually	22 (30.1)
Sometimes	16 (21.9)
Never	0 (0)
During patient counseling, how frequent do you educate patients on: Contraindications*	**Frequency (%)**
Always	27 (37)
Usually	25 (34.2)
Sometimes	20 (27.4)
Never	1 (1.4)

*There were some missing data.

**Fig. 2:**
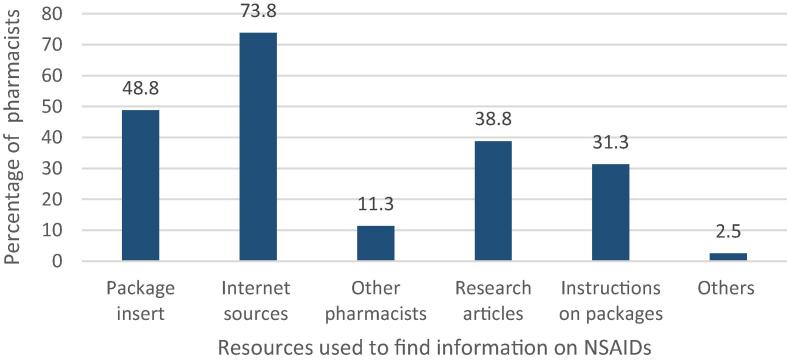
Resources used by community pharmacists in Qatar to find information on NSAIDs (*n* = 80).

Regarding the sources where community pharmacists obtained information on NSAIDs, most of the community pharmacists working in Qatar (59/80, 73.8%) stated that they usually use internet sources for information on drugs, followed by the drug company’s package insert (39/80, 48.8%), and research articles (31/80, 38.8%) respectively. Fig. 2 illustrates the sources used by community pharmacists to know more about NSAID use.

Finally, there was one practice-related question regarding the least important information on NSAIDs that pharmacists should educate patients on during patient counseling. Most of the pharmacists (46/72, 63.9%) selected nonprescription status of pain relievers, followed by counseling the patient to ask a doctor or pharmacist about the safety of adding a new medication while using NSAIDs (13/72, 18.1%). Only 6.9% (5/72) of the pharmacists selected the item related to counseling patient that NSAIDs may be harmful in those at risk of kidney injury because of its harmful effect of lowering blood supply to the kidney.

Table 5 presents community pharmacists’ opinions on the best protective approaches and measures to use for minimizing the GI and renal-associated adverse effects of NSAIDs. The majority of the community pharmacists (55/80, 68.8%) thought that offering a gastroprotective agent to patients is the best protective measure against GI adverse effects. While for the renal protective agents, there was a subtle difference between the following options: changing to a safer drug on the kidney (e.g. acetaminophen), and advising patients not to use NSAIDs for more than 10 days for pain relief and not more than three days to relief fever (49/80, 61.3%, and 50/80, 62.5% respectively).

**Table 5:** Community pharmacists’ opinion on the best measures to use for minimizing gastrointestinal and renal side effects of NSAIDs (*n* = 80).

1- Approaches to minimizing GI side effects of NSAIDs	n (%)
Reducing the dose of NSAIDs	35 (43.8)
Changing to a safer drug on the GI tract (e.g. COX-2 selective inhibitor)	37 (46.3)
Offering a gastro protective agent (e.g. antacids, proton pump inhibitors, histamine receptor blockers, misoprostol)	55 (68.8)
Advising patients to take oral NSAIDs with food or with a full glass of milk/water	21 (26.3)

2- Approaches to minimizing renal side effects of NSAIDs	n (%)

Reducing the dose of NSAIDs	39 (48.8)
Changing to a safer drug on the kidney (e.g. acetaminophen)	49 (61.3)
Advising patients not to use NSAIDs for more than 10 days to relieve pain and not more than 3 days to relieve fever	50 (62.5)
Advising patients to take oral NSAIDs with food or with glass of milk/water	20 (25)

COX-2, cyclooxygenase-2; GI, gastrointestinal; NSAIDs, nonsteroidal anti-inflammatory drugs.

### 3.5 Attitudes of community pharmacists towards today's practice in relation to counseling on the safe use of NSAIDs to reduce the risk of renal and GI injuries

Of the respondents, 74.6% (53/71) believed that their current knowledge on NSAIDs is sufficient to allow them to advice patients on its safe use. Conversely, 14.1% (10/71) did not believe that their current knowledge suffices for them to educate patients on the safe use of NSAIDs, while 11.3% (8/71) do not know if their knowledge on NSAIDs is adequate.

Approximately 94.4% (67/71) of the respondents believed that community pharmacists have a critical role to play in preventing NSAIDs users from the associated kidney and GI adverse effects of this class of agents. More than two-thirds (67.6%, 48/71) of the respondents, believed that today’s practice in Qatar in relation to counselling patients on the appropriate use of NSAIDs has to undergo some changes to ensure the prevention of the kidney and GI adverse effects. Whereas, 18.3% (13/71) of the community pharmacists who responded to this question did not believe that any changes should be implemented in current practice, and 14.1% (10/71) did not know whether current practice needs to undergo changes or not.

Those who believed that current practice has to change were asked to answer an open-ended question that asked them to mention the changes they want to see in practice. They responded with the answers that revolved around the following themes: offering education, CPPD workshops, free conferences, free access to published articles, and free online sessions to community pharmacists on NSAIDs appropriate usage at least two times per year for updating. In addition to the need to change the legislation in that, not all NSAIDs concentrations shall be nonprescription. Moreover, community pharmacists shall conduct prior interview with the patient to identify risk groups and a proper counselling shall be done after the dispensary of NSAID when appropriate. Additionally, the re-evaluation of community pharmacists’ role by the Ministry of Health, and objective structured clinical examination (OSCE) training is demanded for community pharmacists.

**Fig. 3:**
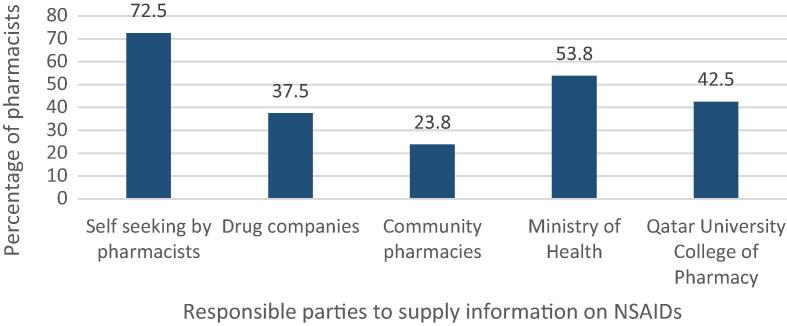
Community pharmacists thoughts on the responsible parties for supplying them with information on NSAIDs (*n* = 80).

Table 6 presents the attitudes of the community pharmacists’ towards the use of NSAIDs in the state of Qatar. Community pharmacists indicated their extent of agreement with five items assessed on a 5-point Likert scale (i.e., strongly agree, agree, neutral, disagree, and strongly disagree). About the statement “It is every patient’s right to be educated on kidney, GI and other adverse effects of NSAIDs”, 95.7% (68/71) of the respondents agreed (strongly agree and agree) with the item. A substantial proportion of community pharmacists (28/70, 40%) were in disagreement (strongly disagreed or disagreed) with the following statement “Provision of information on kidney and gastrointestinal side effects of NSAIDs to patients might be time consuming.” Around 92.8% (65/70) of community pharmacists agreed (strong agreed or agreed) with the following statement “In order to avoid dispensing NSAIDs to high risk patents, pharmacists should ask patients about their health problems and concomitant medication use.” When asked about indicating who is responsible to provide information on NSAIDs to community pharmacists in Qatar, the vast majority of the respondents (51.8%) thought that pharmacists should seek such information on their own. Fig. 3 provides the details of the respondents’ choices on indicating who is responsible to supply pharmacists working in community pharmacies with education on NSAIDs.

**Table 6:** Community pharmacists’ attitudes towards NSAIDs use in Qatar (*n* = 80).

It is every patient’s right to be educated on kidney, gastrointestinal and other side effects of NSAIDs.*	**Frequency (%)**
Strongly agree	41 (57.7)
Agree	27 (38)
Neutral	2 (2.8)
Disagree	1 (1.4)
Strongly disagree	0 (0)
Provision of information on kidney and gastrointestinal side effects of NSAIDs to patients might be time consuming.*	**Frequency (%)**
Strongly agree	11(15.7)
Agree	21 (30)
Neutral	10 (14.3)
Disagree	21 (30)
Strongly disagree	7 (10)
In order to avoid dispensing NSAIDs to high-risk patients, pharmacists should ask patients about their health problems and concomitant medication use.*	**Frequency (%)**
Strongly agree	47 (67.1)
Agree	18 (25.7)
Neutral	3 (4.3)
Disagree	1 (1.4)
Strongly disagree	1 (1.4)

*Some data are missing.

## 4 Discussion

This cross-sectional questionnaire-based study to explore Qatar community pharmacists’ knowledge and attitudes on the GI and renal adverse effects of OTC NSAIDs showed that approximately 90% of the community pharmacists have at least good knowledge on the adverse effects of the medication class. In addition, more than half of the pharmacists educated patients on the dosage, administration, side effects, precautions and contraindications of NSAIDs during their routine practices. Similarly, the majority of the pharmacists have positive attitudes towards educating patients about adverse effects of NSAIDs and identifying high-risk patients who should avoid OTC NSAIDs. However, almost half (45.7%) of the pharmacists strongly agreed or agreed that educating patients about NSAIDs can be time consuming. The community pharmacists indicated the need for continuing education programs at least twice a year to update their knowledge on NSAIDs and the changes in legislation to classify some NSAIDs as prescription only medicines.

A large proportion of the community pharmacists in Qatar demonstrated they are knowledgeable about the adverse effects of NSAIDs. This result is important for the safety of patients and the public in Qatar that utilize the services of community pharmacists, because a knowledgeable pharmacist will properly educate patients on steps to prevent or minimize adverse effects that could sometimes be fatal. To our knowledge, there is no other study conducted to evaluate the knowledge and practices of community pharmacists about the adverse effects of NSAIDs. A study among physiotherapists in Australia reported a similarly high percentage of physiotherapists with good knowledge on NSAIDs’ adverse effects (Grimmer et al, 2002). Nevertheless, some studies that have evaluated the general knowledge of pharmacists in making recommendations about NSAIDs for managing acute or chronic pain in patients with or without comorbidities have suggested that pharmacists may benefit from more training to increase their knowledge and confidence in making appropriate recommendations to patients (Silcock et al., 2007, Ipingbemi, 2016). Therefore, it is not surprising that community pharmacists in Qatar requested for continuing pharmacy education activities at least twice a year to update their knowledge on NSAIDs. The interim solution to this request by community pharmacists will be to access online continuing education provided by reputable educational or professional organizations. Adequate community pharmacists’ knowledge on NSAIDs will ultimately result in safer use due to appropriate evidence-based medications dispensed to patients accompanied with the provision of proper counseling.

Since most NSAIDs can be obtained from the community pharmacy in Qatar without a prescription or pharmacist’s consultation, it is important for pharmacists to take proactive role and the initiative to educate patients/customers who obtain these medications from them. Therefore, it is positive that more than half of the pharmacists surveyed reported routinely educating patients about NSAIDs use, despite admitting that it is a time consuming exercise. More importantly, almost all the pharmacists (94.4%) believed that the community pharmacist plays a critical role in protecting NSAIDs users from renal and GI adverse effects. This finding is similar to a cross-sectional study of community pharmacists in Thailand which found that at least 65% of them conducted screening for history of GI adverse effects or renal impairment prior to dispensing NSAIDs, as well as 36.9–63.2% of pharmacists communicating ADR information occasionally or regularly when they dispense NSAIDs (Phueanpinit et al., 2017). In addition, another cross-sectional study on OTC counseling by community pharmacists in Brazil found that 62.3% of the pharmacists communicated the adverse effects of medications to patients (Halila et al., 2015), while only 52.5% regularly did in Saudi Arabia (Malebari et al, 2020). It is possible that time pressure, inadequate staffing, and other factors play a significant role in why a substantial proportion of community pharmacists do not provide education on the harmful effects of NSAIDs whenever they dispense the product.

This study has several limitations, some of which are inherent to all cross-sectional survey type of studies. First, the number of community pharmacists who responded to the questionnaire (*n* = 114) was lower than the estimated sample size (*n* = 255), despite the prolonged data collection period and several reminders sent to eligible participants to complete the survey. The low participation by community pharmacists was most likely related to their fatigue in filling several questionnaires from different research groups in Qatar. Moreover, not all the three composites of the survey were completed by all the respondents. There is a possibility of type 2 error in the inferential analyses where none of the demographic and clinical characteristics of the pharmacists significantly influenced the knowledge score, because the minimum sample size was not reached. Nevertheless, it is assuring that both the overall mean and median scores fell in the range of good knowledge. There is also the possibility that the community pharmacists who completed the survey have particular interest in the research topic. Therefore, readers should interpret the results with caution as the responding community pharmacists may not be representative of the knowledge of all community pharmacists in Qatar. Another limitation of the study is the use of a convenient sample of pharmacists. Even though the initial intention was to reach out to all community pharmacists in a government database, pharmacy managers of the three largest chain pharmacies in Qatar were contacted to send the questionnaire link directly to pharmacists in their respective organizations after the initial emailing of the link to community pharmacists in order to boost up the numbers of respondents. Furthermore, it is possible that the study participants accessed drug information resources when completing knowledge-based questions of the survey, which may have led to the high knowledge score. In an ideal community pharmacy practice setting, pharmacists should be encouraged to consult appropriate drug information resources in order to prevent avoidable harm to patients. Therefore, if it is part of the routine practice of Qatar community pharmacists to access appropriate drug information resources during patient care, then it is a good practice to improve patient safety. Finally, social desirability bias cannot be ruled out especially for the composites of the survey related to current practices on proper use of NSAIDs and attitudes towards NSAIDs use. However, administering the survey online and assuring respondents that their responses will be kept anonymous hopefully minimized this bias in the study. Therefore, readers should take the possibility of social desirability bias into consideration when interpreting the results of the study.

Future studies evaluating the knowledge and attitudes of pharmacists on the GI and renal adverse effects of NSAIDs should include pharmacists working in the outpatient pharmacies of government and private hospitals in Qatar as well as pharmacists working in the primary health centers. These pharmacists dispense NSAIDs to many patients who receive primary, secondary and tertiary care services in hospitals and health centers. Therefore, it is important to assess the knowledge and attitudes of these pharmacists who dispense NSAIDs to patients who usually have other comorbidities, warranting the need of multiple medications in addition to the NSAIDs.

## 5 Conclusion

Almost two-thirds of the community pharmacists in Qatar who responded to this survey have at least good knowledge regarding the renal and GI adverse effects of NSAIDs. They also reported positive attitudes towards protecting patients against the renal and GI adverse effects of NSAIDs, and this could be reinforced by the completion of CPD programs on NSAIDs once or twice each year. However, significant proportion of the pharmacists admitted that educating patients on NSAIDs was time consuming. This study provides evidence that community pharmacists in Qatar are knowledgeable about the renal and GI adverse effects of NSAIDs and routinely educate patients and customers about safe use of NSAIDs. Ultimately, through patient counselling, the community pharmacists are playing a vital role to prevent or minimize adverse effects from NSAIDs which are widely used in Qatar. Thus, pharmacy managers should give sufficient time to pharmacists to interact with patients and customers who obtain NSAIDs from their respective pharmacies. In addition, we suggest the Ministry of Public Health of Qatar should consider making counseling on high-risk medications (e.g., NSAIDs and insulin compulsory) by community pharmacists mandatory so that measures can be put in place in the pharmacies to free the pharmacist for education and counselling.

## Funding

This study was funded by Qatar University under the Student Grant number QUST-CPH-SPR\2017-14.

## Declaration of Competing Interest

The authors declare that they have no known competing financial interests or personal relationships that could have appeared to influence the work reported in this paper.

## References

[b0005] Bener A., Dafeeah E.E., Alnaqbi K., Falah O., Aljuhaisi T., Sadeeq A., Khan S., Schlogl J. (2013). An epidemiologic analysis of low back pain in primary care: a hot humid country and global comparison. J. Prim Care Commun. Health.

[b0010] Grimmer K., Kumar S., Gilbert A., Milanese S. (2002). Non-steroidal anti-inflammatory drugs (NSAIDs): physiotherapists' use, knowledge and attitudes. Aust. J. Physiother..

[b0015] Halila G.C., Junior E.H., Otuki M.F., Correr C. (2015). Pharm. Pract. (Granada)..

[b0020] Harirforoosh S., Asghar W., Jamali F. (2013). Adverse effects of nonsteroidal antiinflammatory drugs: An update of gastrointestinal, cardiovascular and renal complications. J. Pharm. Pharm. Sci..

[b0025] Ibanez-Cuevas V., Lopez-Briz E., Guardiola-Chorro M.T., on behalf of the NSAID induced Gastropathy Prevention Programme Group (2008). Pharmacist intervention reduces gastropathy risk in patients using NSAIDs. Pharm. World Sci..

[b0030] Institute for Safe Medication Practices, 2007. Protecting U.S. Citizens from Inappropriate Medication Use. A White Paper on Medication Safety in the U.S. and the Role of Community Pharmacists. https://communitypharmacyfoundation.org/docs/CPF_Doc_312222.pdf.

[b0035] Ipingbemi A. (2016). Assessment of pharmacist's knowledge on use of non-steroidal anti-inflammatory drugs in hypertensive patients with co-morbid arthritis and pharmaceutical services rendered in Ibadan, Nigeria. West Afr. J. Pharm..

[b0040] Jang S.M., Cerulli J., Grabe D.W., Fox C., Vassalotti J.A., Prokopienko A.J., Pai A.B. (2014). NSAID-avoidance education in community pharmacies for patients at high risk for acute kidney injury, Upstate New York, 2011. Prev. Chronic Dis..

[b0045] Kaufman D.W., Kelly J.P., Battista D.R., Malone M.K., Weinstein R.B., Shiffman S. (2018). Exceeding the daily dosing limit of nonsteroidal anti-inflammatory drugs among ibuprofen users. Pharmacoepidemiol. Drug Saf..

[b0050] Malebari A.M., Khayyat A.N., Mahdali R.A., Alamoudi J.S., Alsayed B.Y., Alrasheed S.A. (2020). Evaluation of the community pharmacists’ performance in the screening of non-steroidal anti-inflammatory drugs risks in Saudi Arabia. Saudi Med. J..

[b0055] Nielsen National Household Panel, Period of 52 weeks ending January 2018. https://www.nielsen.com/wp-content/uploads/sites/3/2019/04/december-2018-total-consumer-report.pdf.

[b0060] Oliviu, V., 2017. Adverse effects and drug interactions of the non‐steroidal anti‐inflammatory drugs, Nonsteroidal Anti-Inflammatory Drugs, Ali Gamal Ahmed Al-kaf, IntechOpen, DOI: 10.5772/intechopen.68198. Available from: https://www.intechopen.com/books/nonsteroidal-anti-inflammatory-drugs/adverse-effects-and-drug-interactions-of-the-non-steroidal-anti-inflammatory-drugs.

[b0065] Pai A.B. (2015). Keeping kidneys safe: The pharmacist's role in NSAID avoidance in high-risk patients. J. Am. Pharm. Assoc..

[b0070] Phueanpinit P., Pongwecharak J., Sumanont S., Krska J., Jarernsiripornkul N. (2017). Physicians' communication of risks from non-steroidal anti-inflammatory drugs and attitude towards providing adverse drug reaction information to patients. J Eval Clin Pract..

[b0075] Phueanpinit P., Pongwecharak J., Krska J., Jarernsiripornkul N. (2018). Evaluation of community pharmacists’ roles in screening and communication of risks about non-steroidal anti-inflammatory drugs in Thailand. Evaluation of community pharmacists’ roles in screening and communication of risks about non-steroidal anti-inflammatory drugs in Thailand. Primary Health Care Res. Develop..

[b0080] Raosoft® Sample Size Calculator. Available online at http://www.raosoft.com/samplesize.html (accessed September 30, 2016).

[b0085] Silcock J., Moffett J.K., Edmondson H., Waddell G., Burton A.K. (2007). Do community pharmacists have the attitudes and knowledge to support evidence based self-management of low back pain?. BMC Musculoskeletal Disorders.

[b9000] Teichert M., Griens F., Buijs E., Wensing M., De Smet P.A.G.M. (2014). Effectiveness of interventions by community pharmacists to reduce risk of gastrointestinal side effects in nonselective nonsteroidal anti-inflammatory drug users. Pharmacoepidemiol. Drug Saf..

[b0090] Vonkeman H.E., van de Laar M.A.F.J. (2010). Nonsteroidal anti-inflammatory drugs: Adverse effects and their prevention. Semin. Arthritis Rheum..

[b0095] Wilcox C.M., Cryer B., Triadafilopoulos G. (2005). Patterns of use and public perception of over-the-counter pain relievers: focus on nonsteroidal antiinflammatory drugs. J. Rheumatol..

