# Supplementary material for: Assessment of Qatar community pharmacists’ competence and practices related to renal and gastrointestinal adverse effects of nonprescription NSAIDs
Source: Saudi Pharm J. 2022 Jun 22;30(10):1396–404. doi: 10.1016/j.jsps.2022.06.011 (PMC9649341; doi:10.1016/j.jsps.2022.06.011)
Supplement: Supplementary data 1 [file mmc1.docx]

**Appendix**

**Questionnaire**

**Dear Participant:**

Thank you for agreeing to participate in this survey. This survey is part of a study that aims at assessing community pharmacists’ knowledge regarding nonprescription non-steroidal anti-inflammatory drugs (NSAIDs) in Qatar. This questionnaire consists of 28 questions divided into four sections: Demographics, current knowledge on NSAIDs side effects, current practice on proper use of NSAIDs, and attitudes of community pharmacists in Qatar towards NSAIDs use.

Please note that your answers will be anonymous, so no one will be able to find out your identity.

It is completely voluntary to fill out this questionnaire.

It should take you about 10-15 minutes to complete all the questions.

**Please answer all questions thoughtfully. Your answers will contribute in future plans to improve practice.**

**Section 1: Demographics**

**In this section, we would like to gather general information about you.**

1. **What is your gender?**

- Male
- Female

1. **What is your age?**

- <30
- 30-39
- 40-50
- >50

1. **What is your nationality?**

- GCC country (Qatar, Oman, Bahrain, Emirates, Saudi Arabia, Kuwait)
- Middle Eastern country other than GCC (Jordan, Palestine, Lebanon, Syria, Iraq, Yemen)
- North African country (Tunisia, Egypt, Sudan, Morocco, Algeria, Libya)
- Asian country (Iran, Pakistan, India, Bangladesh, The Philippines, etc.)
- Other (please specify): ___________________________

**4. Which community pharmacy are you working at currently?**

- Wellcare
- Kulud
- Ebn Sina
- Other (please specify): _________________________

**5. How long have you been working as a licensed community pharmacist in Qatar:**

- <5 years
- 5-10 years
- >10 years

**6. Highest degree in pharmacy:**

- Bachelor’s degree
- Master’s degree
- Doctor of Pharmacy
- Other (please specify): _______________________

**Section 2: Current knowledge on NSAIDs induced kidney and gastrointestinal injuries**

**In this section, there are 10 questions that will test your general knowledge on NSAIDs and more importantly your knowledge on their kidney and gastrointestinal side effects. Please answer the following questions without using any resources or discussing the questions with other pharmacists.**

**7. NSAIDs are indicated in all of the following conditions except: (choose ONE answer only)**

- Pain
- Acute gout
- Chronic gout
- Headache

**8. NSAIDs interact with all of the following drugs except: (choose ONE answer only)**

- Diuretics
- Clopidogrel
- Warfarin
- Albuterol

**9. NSAIDs are contraindicated in all of the following conditions except: (choose ONE answer only)**

- History of gastrointestinal bleeding
- Asthma
- Hypersensitivity to Ibuprofen
- Osteoporosis

**10. Which of the following is a gastrointestinal side effect of NSAIDs: (choose ONE answer only)**

- Upper gastrointestinal bleeding
- Inflammatory bowel disease
- Irritable bowel syndrome
- Crohn’s disease

**11. Unlike systemic NSAIDs, topical NSAIDs have lower risk of causing epithelial injury in the gastrointestinal tract:**

- True
- False
- I do not know

1. **Inhibition of prostaglandin synthesis causes: (Choose ONE answer only)**

- Decreased gastric acid secretion, increased bicarbonate secretion and increased mucus secretion.
- Increased gastric acid secretion, decreased bicarbonate secretion and decreased mucus secretion.
- None of the above.

**13. Risk factors for developing gastric injury include all of the following except: (choose ONE answer only):**

- High dose of NSAID
- Previous medical history of gastrointestinal disease (e.g. ulcers)
- Age less than 65 years
- Concomitant steroid therapy of 10 mg/day or greater

**14. The risk of NSAID-induced kidney injury increases with: (choose ONE answer only)**

- Lower NSAID doses
- Concomitant use of ACE inhibitors
- Shorter duration of NSAID therapy
- Concomitant use of topical capsaicin

**15. All the following patients should avoid NSAIDs except: (choose ONE answer only)**

- Patients having a higher risk of stomach bleeding.
- Patients having stomach problems, including heartburn.
- Patients having high blood pressure, heart disease, liver cirrhosis, or kidney disease.
- Patients with only hypothyroidism, for which they take levothyroxine rally
- Patients taking a diuretic for high blood pressure or heart failure

**16. Which of the following is the most appropriate alternative for a patient at high risk for NSAID induced acute kidney injury after an acute musculoskeletal injury? (choose ONE answer only)**

- Naproxen
- Capsaicin cream
- Acetaminophen/Paracetamol
- Colchicine

**Section 3: Current practice on NSAIDs proper use**

**In this section, we would like to evaluate the current practice in community pharmacies in relation to educating patients on the safe use of NSAIDs and the measures used to minimize associated side effects.**

**17. When did you last update your knowledge on NSAIDs? (E.g. attended a conference on NSAIDs, read articles on NSAIDs)**

- I did not update my knowledge on NSAIDs
- < 1 year ago
- 1-5 years ago
- 6-10 years ago
- > 10 years ago
- I do not recall

**18. If you have updated your knowledge on NSAIDs then, how did you do so?** (Tick ✔ as many as you require)

- Via another community pharmacist
- Research articles
- Internet
- Continued education program (e.g. Continuing Professional Development Program of Healthcare Practitioners “CPD-HCP”)
- Attending a conference on NSAIDs
- Drug information books
- Other (please specify): _______________________

1. **Do you commonly recommend NSAIDs to the customers who visit your community pharmacy?**

- Yes
- No

1. **Which NSAID do you usually recommend? (choose ONE answer only)**

- Ibuprofen
- Naproxen
- Diclofenac
- Aspirin
- Other (please specify): _______________________

1. **During patient counseling, how frequent do you educate patients on**

| **Statement** | **Always** | **Usually** | **Sometimes** | **Never** |
| --- | --- | --- | --- | --- |
| Instructions on taking the medicine |  |  |  |  |
| Dosage |  |  |  |  |
| Side effects and precautions |  |  |  |  |
| Contraindications |  |  |  |  |

1. **In your opinion which of the following measures or approaches are best to use for minimizing gastrointestinal side effects of NSAIDs: (Check as many as you require)**

- Reducing the dose of NSAIDs
- Changing to a safer drug on the gastrointestinal tract (e.g. COX2 selective inhibitor )
- Offering a gastro protective agent (e.g. Antacid, Proton pump inhibitors, Histamine receptor blockers, Misoprostol )
- Advising patients to take oral NSAIDs with food or with a full glass of water

1. **In your opinion which of the following measures or approaches are best to use for minimizing the renal side effects of NSAIDs: (Check as many as you require)**

- Reducing the dose
- Changing to a safer drug on the kidney (e.g. Acetaminophen )
- Advising patients not to use NSAIDs for more than 10 days to relief pain and not more than 3 days to relief fever
- Advising patients to take oral NSAIDs with food

**24. Which of the following sources do you usually use to find out more about NSAIDs? (Check as many as you require)**

- Drug company’s package insert
- Internet sources on drugs
- Other Pharmacists
- Research articles
- Instructions on packets
- Other (please specify): ______________________
- None

**25. Which of the following is the least important teaching point on NSAID to counsel patients on? (choose ONE answer only)**

- NSAIDs are nonprescription pain relievers in Qatar.
- Before using any medicine ask your doctor or pharmacist if it is safe to use it while using NSAIDs, including nonprescription medicines, vitamins, and herbal products
- NSAIDs may not be good in people at risk for kidney disease because they may harm the kidneys by lowering blood supply to the kidney.
- Adding NSAIDs to some blood pressure medicines can increase the possibility of harm to the kidney through decreased blood flow

**Section 4: Beliefs towards NSAIDs use in Qatar**

**In this section, we would like to explore your beliefs in relation to counseling patients on NSAIDs-related kidney and gastrointestinal side effects and the importance of community pharmacists’ role in today’s practice.**

**26. Do you believe that:**

| **Statement** | **Yes** | **No** | **I do not know** |
| --- | --- | --- | --- |
| Your current knowledge of NSAIDs is sufficient to allow you to advise patients on safe use. |  |  |  |
| Community pharmacists have a critical role in preventing NSAIDs related kidney and gastrointestinal injuries. |  |  |  |
| 1. Today’s practice in Qatar in relation to counseling on appropriate use of NSAIDs needs to undergo some changes in order to prevent kidney and gastrointestinal side effects. |  |  |  |
| If your answer to statement three is yes, then what do you think shall be changed or implemented in current practice? |  | | |

**27. Please indicate your level of agreement with the following statements by ticking the appropriate box. (Choose only ONE option for each statement)**

| **Statement** | **Strongly agree** | **Agree** | **Neutral** | **Disagree** | **Strongly disagree** |
| --- | --- | --- | --- | --- | --- |
| It is every patient’s right to be educated on kidney, gastrointestinal and other side effects of NSAIDs. |  |  |  |  |  |
| Provision of information on kidney and gastrointestinal side effects of NSAIDs to patients might be time consuming. |  |  |  |  |  |
| In order to avoid dispensing NSAIDs to high risk patents, pharmacists should ask patents about their health problems and concomitant medication use. |  |  |  |  |  |

**28. Whose responsibility do you think it is to provide information on NSAIDs to community pharmacists in Qatar?**

(Check as many as you require)

- Pharmacists should seek it themselves
- Drug companies
- Community pharmacies owners/coordinators
- Ministry of health
- College of pharmacy at Qatar university
- Other (please specify) : ____________________________________________

**Thank you for taking the time to complete this questionnaire.**
